# Understanding Influenza A(H3N2) Subclade K (J.2.4.1): Asian Epidemiology, Clinical Features, and Public Communication Challenges

**DOI:** 10.1111/irv.70240

**Published:** 2026-02-09

**Authors:** Timotius Ivan Hariyanto

**Affiliations:** ^1^ Department of Microbiology Faculty of Medicine, Universitas Airlangga Surabaya Jawa Timur Indonesia

Dear Editor,

1

Since mid‐2025, a newly designated variant of seasonal influenza A(H3N2), commonly referred to in sequence nomenclature as J.2.4.1 and aliased by Nextstrain as subclade K, has attracted intense attention from clinicians, virologists, and the public [1]. Genetically, subclade K differs from immediately preceding H3N2 viruses by a constellation of amino acid substitutions clustered in the haemagglutinin (HA) surface glycoprotein (notably T135K, K189R, and N158D among others), changes that alter antigenic sites and have produced reduced reactivity with ferret antisera raised against the 2025 vaccine strain [1]. These molecular shifts explain why subclade K is being monitored as a drifted H3N2 lineage rather than being treated as a fundamentally novel pathogen [1].

Global genomic surveillance indicates that influenza A(H3N2) subclade K increased rapidly in frequency from August to September 2025 and, by late 2025, had been detected across most continents, consistent with widespread dissemination rather than localized spread [2]. GISAID sequence metadata as of January 13, 2026 further show that subclade K is now well established in Asia, with clear signals of recent expansion [2]. The largest cumulative numbers of reported sequences or isolates were recorded in Thailand (229), followed by China (151), Korea (135), and Japan (113), reflecting substantial circulation in both East and Southeast Asia [2]. Importantly, recent reporting dominated in several settings: Korea documented 103 of 135 detections (96.3%) within the past 4 weeks, while Thailand (153/229; 62.2%), Japan (95/113; 60.5%), and China (135/151; 45.6%) also showed a high proportion of recent detections, consistent with rapid lineage replacement [2]. Although absolute counts were lower, Malaysia, Cambodia, Vietnam, Indonesia, and Brunei each reported that more than 80% of their cumulative detections occurred in the most recent 4‐week period, suggesting recent introduction or intensified surveillance [2]. By contrast, India reported limited cumulative detections without recent additions, highlighting regional heterogeneity in transmission and sequencing activity [2]. Overall, the predominance of recent detections across multiple Asian countries supports ongoing expansion of subclade K, paralleling observations from the southern hemisphere in 2025, where its emergence coincided with prolonged influenza seasons [3].

Clinically, infections attributed to subclade K present with the spectrum of illness typical for seasonal influenza: abrupt onset of fever, cough, myalgia, sore throat, nasal congestion, and malaise; severe outcomes continue to cluster among older adults, infants, pregnant people, and those with chronic comorbidities [1, 4]. To date, aggregated epidemiological summaries from WHO and European public health agencies have not demonstrated a consistent signal for greater intrinsic virulence of subclade K compared with other contemporary H3N2 viruses, though the public health impact can be amplified by high case numbers and partial vaccine mismatch [1, 4].

Diagnostic testing relies on the same tools used for standard seasonal influenza: nucleic acid amplification tests (NAAT/PCR) for confirmation and subtyping, with rapid antigen tests used in many clinical settings for point‐of‐care decisions [1, 4]. Where needed for surveillance or research, sequencing identifies subclade K genomes and informs antigenic and phylogenetic interpretation [1, 4]. Management follows established influenza clinical practice guidelines: supportive care for most patients, and early antiviral therapy (oseltamivir, peramivir, zanamivir, or baloxavir, where available and indicated) for high‐risk or severely ill patients—ideally begun within 48 h of symptom onset to maximize benefit [1, 4]. Public health measures (vaccination, respiratory hygiene, voluntary isolation while symptomatic, and appropriate use of masks in high‐risk settings) remain primary prevention tools [1, 4].

Because subclade K contains antigenic changes relative to the H3N2 component selected for the 2025–2026 northern hemisphere vaccine, laboratory assays have shown reduced serologic reactivity of K viruses to vaccine strain antisera in some early reports [5]. Real‐world vaccine effectiveness (VE) analyses are heterogeneous: Observational VE estimates from early surveillance in England and other settings show some retained protection against severe outcomes but lower effectiveness against mild infection for H3N2 subclade K compared with better‐matched seasons [5]. Importantly, even partially matched vaccines can blunt severe disease and reduce hospital pressure; immunization remains the most effective population‐level intervention available [5].

Media outlets in several countries have popularized the label “superflu” in headlines about subclade K [6, 7]. The term is evocative and communicates an elevated threat in a single phrase, which has advantages for public readability but carries substantial risks: It may imply an unprecedented pathogenicity, foster panic, or incentivize overutilization of emergency services by low‐risk individuals. Coverage that emphasizes novelty without conveying nuance—that subclade K is a drifted seasonal H3N2 lineage with potential for widespread transmission but not proven increased virulence—can distort public perception and complicate rational public health responses. Analyses by public health communicators and vaccine advocates have noted this tension and urged precise language that distinguishes rapid spread (high incidence) from intrinsic severity.

In contrast, peer‐reviewed scientific communications and formal surveillance reports uniformly use technical names (A(H3N2) subclade J.2.4.1, alias K) and report measured descriptors—genetic mutations, antigenic characterization, vaccine reactivity, VE estimates, and epidemiological metrics—without resorting to sensational labels [3, 5, 8]. Representative scientific pieces, including a recent Perspective in JAMA and surveillance‐based analyses in Eurosurveillance, frame subclade K as an antigenic and epidemiological development that warrants close monitoring and calibrated public health action rather than alarmist rhetoric [3, 5, 8]. This difference—evocative media shorthand versus precise scientific framing—should guide clinicians and public health professionals when communicating with patients and the press.

In conclusion, influenza A(H3N2) subclade K is best understood as a drifted seasonal H3N2 lineage whose spread has had measurable impacts on the timing and intensity of the 2025–2026 influenza season regionally and globally. The principal concerns are its rapid transmissibility and partial antigenic mismatch to the current vaccine strain, not clear evidence of greater virulence. Maintaining high standards of genomic surveillance (GISAID/Nextstrain), clear clinical testing and treatment pathways, and careful, nonsensational public communication will reduce unnecessary alarm while protecting the vulnerable. Health authorities should emphasize vaccination, early testing, and prompt antiviral therapy for at‐risk groups, and media should avoid shorthand such as “superflu” unless clearly contextualized by authoritative scientific evidence.

## Author Contributions

**Timotius Ivan Hariyanto:** conceptualization, investigation, funding acquisition, writing – original draft, writing – review and editing, visualization, validation, methodology, project administration, formal analysis, software, resources, supervision, data curation.

## Funding

The author has nothing to report.

## Ethics Statement

The author has nothing to report.

## Conflicts of Interest

The author declares no conflicts of interest.

## Data Availability

The author confirms that the data supporting the findings of this study are available within the article.
